# Aggressive Bladder Tumour Following Clam Ileocystoplasty: A Rare Long-Term Complication

**DOI:** 10.7759/cureus.48388

**Published:** 2023-11-06

**Authors:** Reginald Ononye, Luke Mistry

**Affiliations:** 1 Urology, Glan Clwyd Hospital, Betsi Cadwaladr University Health Board, Rhyl, GBR

**Keywords:** bladder cancer following clam ileocystoplasty, malignant transformation following bladder augmentation, long term complications of clam ileocystoplasty, surgical treatment of overactive bladder syndrome, oab syndrome, chronic complications of bladder augmentation, complications of bladder augmentation, bladder augmentation, clam ileocystoplasty

## Abstract

Overactive bladder syndrome (OAB) is a common, chronic, complex, often under-reported and under-treated condition with a significant impact on quality of life. It poses a high burden on healthcare systems. Clam ileocystoplasty is an invasive procedure typically reserved for the treatment of severe refractory cases. Malignant transformation in the area of anastomosis remains a rare but real, life-threatening risk in the patient group and requires active life-long surveillance and follow-up. We report the case of a 51-year-old woman who presented with recurrent urinary tract infections and non-visible haematuria 30 years after clam ileocystoplasty for urge incontinence. Imaging revealed an anterior bladder tumour with hepatic metastases. On multiple occasions, she was unfit for any invasive surgical sampling options to obtain tissue samples to allow for treatment planning, and was provided with best supportive care. The disease rapidly progressed to death within 10 weeks of presentation. This case buttresses the need for informed consent regarding the risks, including malignant transformation several years after the procedure, and the necessity for lifelong follow-up and surveillance cystoscopy, with frequency tailored to individual patient risk assessment.

## Introduction

Overactive bladder syndrome (OAB) is a common, chronic, complex, often under-reported and under-treated condition. It has a significant impact on the quality of life of sufferers and incurs enormous healthcare costs [1,2]. Bladder augmentation as a treatment for OAB syndrome is now rarely done, with the advent of botulinum therapy and other less invasive procedures [3,4], but remains an important third-line option [1]. In the mid-20th century, it was widely carried out to treat the long-term complication of tuberculous cystitis; a small, contracted and unstable bladder [5,6]. More recently, it is mainly indicated in severe refractory OAB, especially of neurogenic origin [7-11].

Clam ileocystoplasty involves using a segment of the ileum retaining its mesentery, divided at the antimesenteric border and anastomosed to the superior aspect of the bladder, resulting in a more capacious bladder, and improving the symptoms of detrusor overactivity and instability [4,5,11]. Clam ileocystoplasty, like other bladder augmentation procedures, has been shown to satisfactorily treat refractory detrusor overactivity. However, it can be associated with a variety of complications, including excessive mucus production, the need for regular clean intermittent self-catheterization (CISC), recurrent urinary tract infections (UTI), bladder stones, and late long-term metabolic and bowel-related problems [3,8,9,11].

Malignant transformation along or close to the anastomosis following clam ileocystoplasty is a rare (incidence of 1 in 250) [4] but feared long-term complication. It follows a long latency period of 10-15 years and is frequently advanced or metastatic at the time of diagnosis [5,6], necessitating a high index of suspicion and surveillance for early diagnosis. The timing and conduct of surveillance cystoscopy remain a topic of debate [12].

This paper reports a case of an aggressive bladder cancer 30 years following clam ileocystoplasty for OAB, urge incontinence and enuresis, it provides insights into the importance of life-long follow-up, keeping a high index of suspicion and surveillance cystoscopy. Physicians need to be vigilant, maintaining a high index of clinical suspicion, even in cases of patient-reported symptoms, to facilitate prompt diagnosis and subsequent treatment [13].

## Case presentation

Background and presentation

A 51-year-old lady known to the urology department was referred by her GP to the Rapid Access Cancer Clinic (RACC) for recurrent UTI and microscopic haematuria. On consultation, she had symptomatic UTI and urethral stenosis. She was commenced on a course of antibiotics and planned for urgent urethral dilatation and cystoscopy under general anaesthesia. Her medical history was significant for obesity, controlled hypertension, vitamin B12 deficiency, depression, and low back pain. She underwent clam ileocystoplasty 30 years prior (early ‘90s) for intractable urge incontinence and enuresis, then left ureteric reimplantation five years later on account of significant vesicoureteral reflux. She had a satisfactory outcome post-op - regained continence, needing intermittent self-catheterization (ISC) four to six times a week, preserved renal function, and no metabolic or bowel dysfunction.

She was seen 10 years earlier with worsening procedure-related complications - significant mucus production requiring regular bladder wash-out, and she resumed regular ISC, she developed a long history of recurrent UTI necessitating several courses of therapeutic and prophylactic antibiotics, and urethral stenosis. She started annual surveillance check cystoscopy with bladder biopsies 10 years post-op but was lost to follow-up during the COVID-19 pandemic outbreak, three years before referral.

Outcome

**Figure 1:**
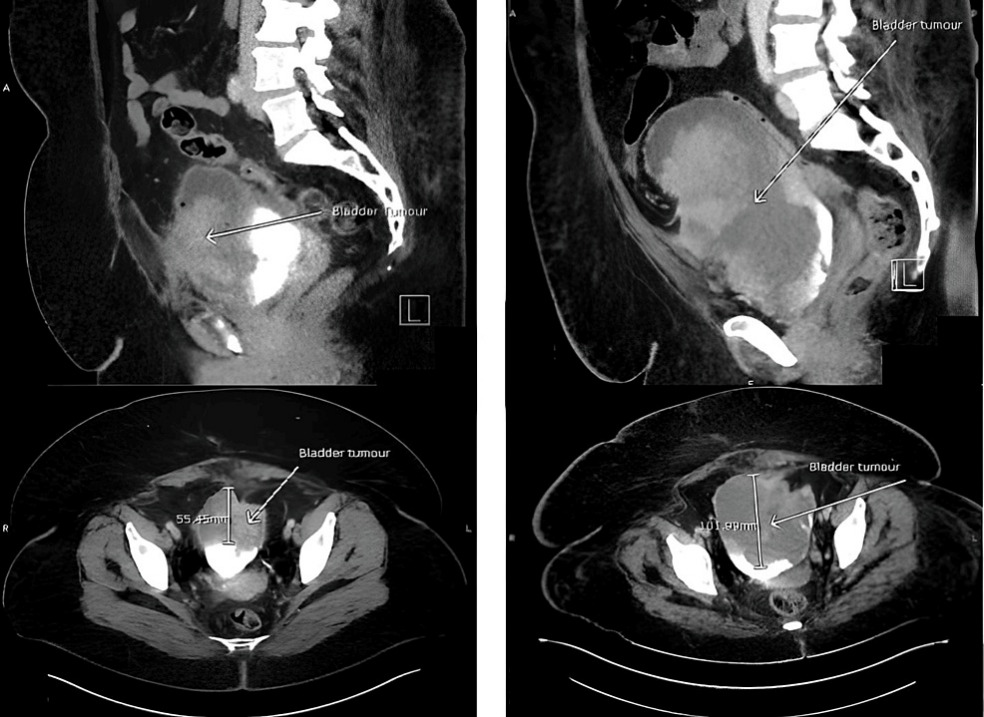
Comparative CT-Urography images showing progression of bladder mass. Cross-sectional images (sagittal and axial) - (Left) at presentation vs (Right) eight weeks later

**Figure 2:**
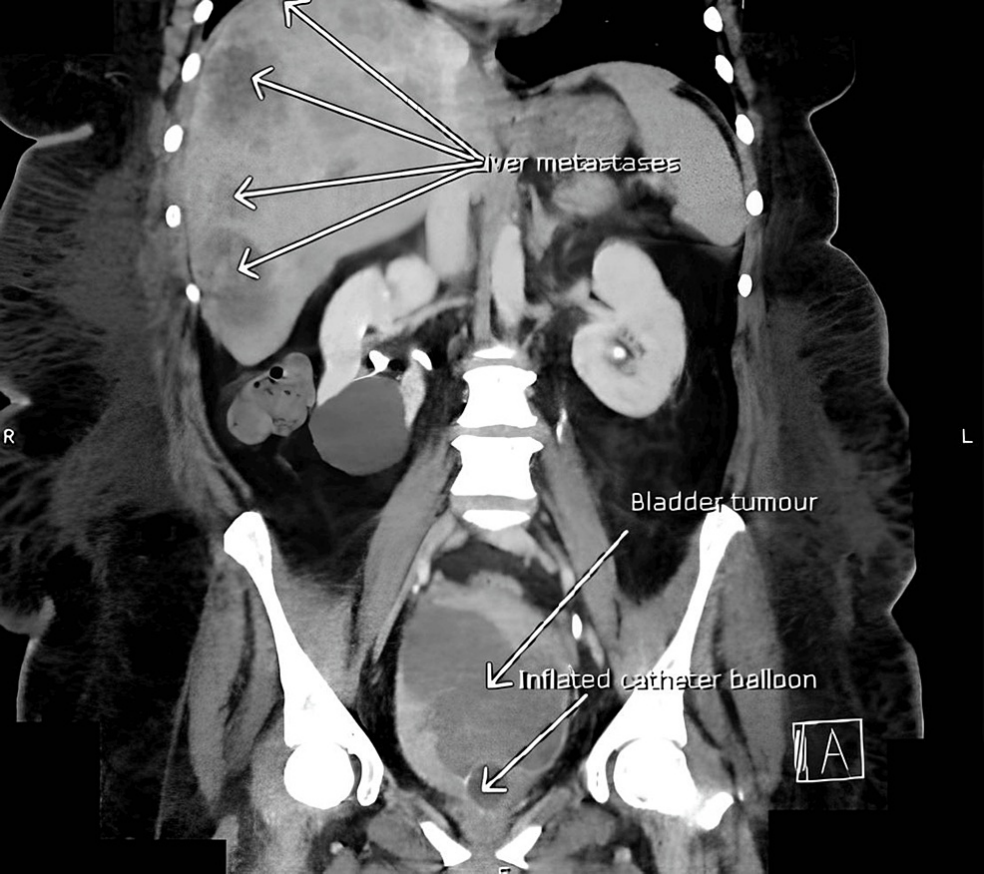
Coronal section from second CT-Urography demonstrating large bladder mass and metastatic liver disease.

Eight days after being seen at the RACC, the patient was admitted via the ED with urosepsis. At presentation, investigations revealed raised inflammatory markers (white cell count 24.5 x 109/L, neutrophils 17. x 109/L, C-reactive protein 277 mg/dL), anaemia (haemoglobin 78 g/L), raised alkaline phosphatase (778 U/L), and normal renal function. She was started on meropenem following consultation with microbiology. Urine culture revealed resistant *Escherichia coli* and *Enterococcus *sp. (see Table 1 for sensitivities). Repeated blood cultures yielded no growth. CT urogram revealed significant findings of a 5.5 cm anterior bladder tumour with hepatic metastases and a repeat three weeks later showed significant disease progression (bladder tumour was now >10 cm with evidence of emphysematous cystitis and increased hepatic disease burden). Adenocarcinoma of the bladder was the top radiological differential within the context of her clinical history (Figure 1 and Figure 2).

**Table 1:** Urine culture sensitivity “S” - Sensitive, “R” - Resistant

Antibiotic	E. coli	Enterococcus
Nitrofurantoin	S	S
Ciprofloxacin	R	
Fosfomycin	S	
Gentamicin	S	
Trimethoprim	R	
Piperacillin/Tazobactam	R	
Amoxicillin	R	S
Cotrimoxazole	R	
Linezolid		S
Tigecycline		S

She was subsequently planned for emergency transurethral resection of the bladder tumour but was unfit for surgery, and her general health continued to deteriorate. The patient remained unwell with repeated, prolonged admissions for urosepsis, delaying the planned cystoscopy and biopsy. Following a uro-oncology multidisciplinary meeting, the consensus was for the best supportive care with palliative intent, and an attempt at transjugular liver biopsy to obtain tissue samples for histological diagnosis. The patient unfortunately continued to deteriorate and ultimately passed away within 10 weeks of the emergency presentation.

Interestingly, a documented encounter with the patient during the period of illness revealed that she understood the bladder augmentation surgery could have contributed to the development of her bladder tumour. She however maintained that her condition before the procedure was so debilitating and greatly impacted her quality of life that she would take her chances with it regardless of risk of malignancy. She only wished the tumour was detected early enough to allow for treatment.

## Discussion

The indications for bladder augmentation procedures have significantly narrowed over recent years. It is reserved for refractory cases of OAB in very carefully selected patients [9]. It is carried out to improve bladder functional capacity, the common goal is to promote urinary drainage into a low-pressure urinary reservoir with adequate capacity [3].

Clam ileocystoplasty has been shown by several retrospective studies and case series to be safe and effective in treating OAB symptoms but associated with early and late complications [6,7], and the National Institute for Health and Care Excellence (NICE) has published guidance for the conduct of the procedure [4]. In the presented case, significant improvements in bladder function were seen, continence was regained post-procedure and remained so until incapacitated by illness.

The procedure can be fraught with troublesome complications, including recurrent urinary tract infections, excessive mucus production, and frequent plugging, which can occur relatively early post-op and persist throughout life. The most serious and life-threatening complication is the rare but real risk of malignant transformation in the area of anastomosis [8,11], which unfortunately led to the demise of the index patient. The tumour was metastatic at presentation, consistent with literature reports [5,6]. The nitrosamine theory has since been proposed as a possible pathogenetic process for malignant transformation [5,12]. It was puzzling however, the rapid progression and aggressiveness of the disease leading to death less than three months from diagnosis. The commonest histological type reported is adenocarcinoma, followed by transitional cell tumours [6,12]. In this case, the tumour was thought to behave like an adenocarcinoma of the bladder - early metastases and poor prognosis, however, a histological diagnosis could not be obtained.

Malignant transformation following clam ileocystoplasty typically presents with visible or nonvisible haematuria 10-15 years post-procedure [5,6], similar to the presentation in the index case. Diagnosis is made via cystoscopic examination, followed by transurethral resection of the bladder tumour - the mainstay of diagnosis [14], and staged radiologically for further treatment planning. In this case, the diagnosis was made via clinical history and imaging, as the patient was unfit for any invasive diagnostic or therapeutic intervention, and consequently, a palliative approach was adopted following the uro-oncological multidisciplinary team meeting.

The importance of clinicians having a high index of suspicion and life-long follow-up cannot be overemphasized. The patient had regular annual cystoscopy until she was lost to follow-up during the COVID-19 pandemic, representing within three years with advanced metastatic bladder cancer.

Learning points

Clam ileocystoplasty, although currently rarely performed, continues to occupy a niche in the treatment of severe refractory neurogenic OAB, and is the most effective at achieving sustained symptom control.

The long-term risk of malignant transformation must be fully explored with patients, in addition to the more common chronic complications.

Every episode of visible/non-visible haematuria in patients with background bladder augmentation must be carefully and fully evaluated with a high index of suspicion.

Life-long follow-up and regular surveillance cystoscopy tailored to individual patients’ needs rather than predetermined intervals could help early detection of malignant transformation and must be emphasised.

## Conclusions

Bladder augmentation continues to play an important role in the treatment of severe, refractory OAB. It is safe, effective, and provides good long-term symptom control, although it is invasive with a long potential list of complications, including malignant transformation. In some centres in the UK, clam ileocystoplasty is currently performed laparoscopically allowing for quicker recovery, and shorter convalescence.

The case emphasises the role of life-long follow-up and regular surveillance cystoscopy to allow for early detection of the rare but established risks of malignant transformation, which can run an aggressive course.
